# An observational study of monitoring of vital signs in children admitted to Kenyan hospitals: an insight into the quality of nursing care?

**DOI:** 10.7189/jogh.08.010409

**Published:** 2018-06

**Authors:** Morris Ogero, Philip Ayieko, Boniface Makone, Thomas Julius, Lucas Malla, Jacquie Oliwa, Grace Irimu, Mike English, on behalf of the Clinical Information Network author group

**Affiliations:** 1 Kenya Medical Research Institute (KEMRI) – Wellcome Trust Research Programme, Nairobi, Kenya; 2 Department of Paediatrics and Child Health, University of Nairobi, Kenya; 3 Nuffield Department of Medicine, University of Oxford, Oxford, UK

## Abstract

**Background:**

Measurement and correct interpretation of vital signs is part of routine clinical care. Repeated measurement enhances early recognition of deterioration, may help prevent morbidity and mortality and is a standard of care in most countries.

**Objective:**

To examine documentation of vital signs by clinicians for admissions to paediatric wards in Kenyan hospitals, to describe monitoring frequency by nurses and explore factors influencing frequency.

**Methods:**

Vital signs information (temperature, respiratory and pulse rate) for the first 48 hours of admission was collected from case records of children admitted with non-surgical conditions to 13 Kenyan county hospitals between September 2013 and April 2016. A mixed effect negative binomial regression model was used to explore whether the severity of illness (indicated by danger signs or severe diagnostic episodes) is associated with increased vital signs observation frequency.

**Results:**

We examined 54 800 admission episodes with an overall mortality 6.1%. Nurse to bed ratios were very low (1:10 to 1:41 across hospitals). Admitting clinicians documented all or no vital signs in 57.0% and 8.4% cases respectively. For respiratory and pulse rates there was pronounced even end-digit preference (an indicator of incorrect information) and high frequency recording of specific values (*P* < 0.001) suggesting approximation. Monitoring frequency was explored in 41 738 children. Those with inpatient stays ≥48 hours were expected to have a vital signs count of 18, hospitals varied but most did not achieve this benchmark (median 9, range 2-30). There were clinically small but significant associations between vital signs count and presence of multiple severe illnesses or presence of severe pallor (adjusted relative risk ratio = 1.04, *P* < 0.01, 95% confidence interval CI = 1.02-1.06 and 1.05, *P* = 0.02, 95% CI = 1.01-1.09, respectively).

**Conclusions:**

Data suggest accurate admission measures are sometimes missing especially for pulse and respiratory rates, possibly linked to manual measurement. Monitoring frequency is often low in the high risk population studied probably indicating how quality of nursing care is undermined by considerable human resource shortages.

Mortality on inpatient paediatric wards is high in low-income countries (LIC) with many deaths occurring within the first days of admission [1,2]. Studies on quality of care in such LIC hospitals suggest there are major opportunities for improvement but most focus on the care provided by medical staff [3,4], much less attention has been paid to the routine care given by nursing staff who are often in short supply [5]. Although debate continues on the appropriate frequency of monitoring [6-11] and what comprises an abnormality in vital signs [12] there is a general consensus that regular patient assessment may help detect deterioration. Studies have indicated that this improves patient safety both in medical and surgical units [13-15] and may reduce morbidity and mortality particularly in more severely ill patients [15-19]. Thus, monitoring vital signs is often considered a key nursing task and often contributes to early warning scores [8,20,21] designed to detect patients who are at risk of deterioration.

Given the potential importance of vital signs measurements and the opportunity such assessment provides for identifying patients requiring more general clinical review, we were interested in:

- The frequency of vital signs monitoring for children admitted to Kenyan public hospitals that vary in their geographic setting and mortality rate.
- Whether there is any evidence of prioritization of vital signs monitoring to those with more severe illness or danger signs in settings with limited human resources.
- Likely validity of vital signs observations.

## METHODS

### Study design and setting

We utilise data from patients admitted to paediatric wards across 13 county (formerly district-level) hospitals in Kenya. A detailed description of the selection of hospitals in this study has been reported elsewhere [1]. In brief, these hospitals were purposefully selected to represent high and low or very low malaria prevalence settings and spanning large urban environments and rural county towns that are likely to admit at least 1000 children per year. Collectively, hospitals in this study are part of the Clinical Information Network (CIN) which is designed as a collaboration between researchers from the KEMRI-Wellcome Trust Research Programme, the Kenya Ministry of Health, the Kenya Paediatric Association and the University of Nairobi. One smaller hospital of the 14 in CIN not designated as a county referral hospital and with no attending physicians was excluded from these analyses as this site has a different complement of nurses and different patient case-mix. A principal aim of the CIN is to use de-identified patient-level data to begin to understand and improve hospital care. The work undertaken by the CIN has received approval from the national Kenya Medical Research Institute scientific and ethical review committees.

### Study population, data collection and management

**Figure 1:**
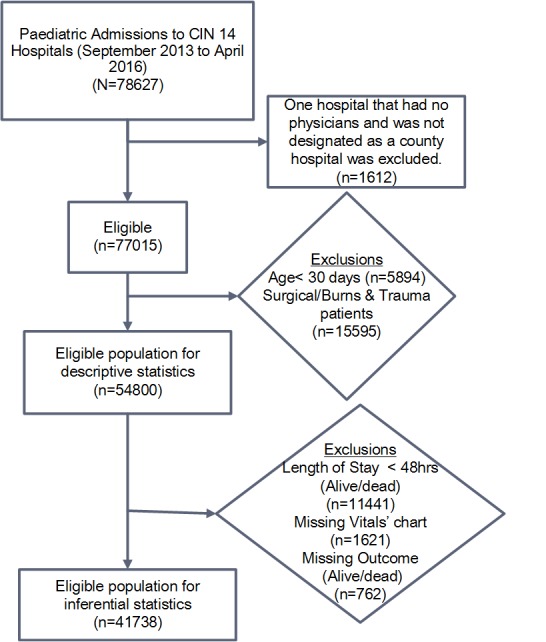
Populations used in different analyses.

All patients aged ≥1 month hospitalized in the paediatric wards of 13 hospitals were eligible for inclusion from September 2013 through April 2016. Patients with surgical conditions or burns were excluded (**Figure 1**). Data were abstracted on discharge or death by a trained data clerk from each of the patient records, aided by clinicians’ use of a Paediatric Admission Record (PAR) form [22], into a customised data capture tool designed in the non-proprietary Research Electronic Data Capture (REDCap, Vanderbilt University, USA; https://www.project-redcap.org/) platform [23]. Data captured and used in these analyses included: basic biodata, history, examination findings, initial vital signs measures recorded in the admitting clinician’s record, diagnosis (defined by the International Classification of Diseases-Tenth Revision (ICD-10) codes), and measurements recorded by nurses on the patient vital signs monitoring chart. The latter data focused on all temperature (T), pulse (P) and respiratory rate (R) measures recorded in the first 48 hours of admission, the time of highest mortality [1]. Data from all 13 hospitals were synchronized to a central server on a daily basis. A full description of the data collection procedures, data quality monitoring, validation framework and the web-based data synchronization is provided elsewhere [24].

### Analysis

We first explored whether an “admission set” of vital signs (T, P, R) were recorded in the medical record by the clinician on duty at admission. The primary outcome for other analyses was the count of subsequent vital signs taken by nurses determined by counting the number of times temperature (T), pulse (P) and respiratory rate (R) were documented on the nursing vital signs chart during the initial 48-hour period. The nurses’ vital signs count was then used in two separate analyses: (a) comparison of the median patient-level count for each hospital with a standard representing the minimum count expected during this initial period of inpatient stay; (b) negative binomial regression analysis to determine the relationship between vital sign (count) and signs of severe illness, admission syndromic diagnosis, child’s age and outcome of admission at patient level. The standard for comparisons of vital signs counts was derived from consensus discussions with senior nurses from the CIN hospitals (collaborators). It was agreed that a reasonable standard, in addition to the ‘admission set’ of clinical signs documented in the medical record, was to have a minimum of 9 observations (3T, 3P and 3R) in 24 hours (representing one set of vital signs observations per nursing shift) and consequently a minimum of 18 observations in 48 hours. As times of admission, death or discharge are not routinely available we stratified patients into different groups. These include those that died on the admission date (within 24 hours) and one or two days after the admission date (Day 1 deaths, approximately 24 hours stay; Day 2 deaths, approximately 48 hours stay). Children who survived the initial 48-hour of admission were further stratified into groups of those with and without danger signs on admission as defined by WHO and Kenyan guidance [25,26].

Data are described using medians, interquartile ranges (IQR), and proportions where appropriate. We use graphical presentations of vital signs values and counts to examine the variability across patient groups and hospitals and Venn diagrams to examine the pattern of admission vital signs recording by clinicians. We used a right-tailed binomial test to test a null hypothesis of no end-digit preference when clinicians record vital signs observations at admission (for example, overall we would expect the number of respiratory rate observations that are even numbers to equal those that are odd numbers, on average).

To explore factors that influence variability of the vital signs count across hospitals, we restricted analyses to patients who had an inpatient stay of at least 48 hours with survival status documented and who had a nursing vital sign chart present in the medical record (n = 41 738, **Figure 1**). We hypothesised *a priori* patient-related factors that might affect frequency of vital signs monitoring in paediatric wards. These factors were based on the clinical judgement of the authors and included: i) patient age group, ii) subsequent survival status (alive/dead, in this case we consider that this later outcome may be a marker for clinicians/nurses ability to recognise factors that provide them with an initial ‘gut feeling’ about severity of patients’ illness [27] that may influence their actions), iii) indicator variables (taking the value 0 or 1) for each admission diagnosis with a ‘severe disease classification’ spanning malaria, pneumonia, anaemia, dehydration, malnutrition, or meningitis (Kenyan and WHO guidelines provide criteria for assigning level of severity [25]) and a distinct indicator variable for those cases with multiple severe illnesses (also coded 0/1), and iv) an indicator variable (taking the value 0 or 1) for each clinical danger sign from those highlighted in Kenyan guidelines including altered level of consciousness (value<A on Alert Voice Pain Unresponsive (AVPU) scale), history of convulsion, or presence of central cyanosis, severe pallor, vomiting everything, acidotic breathing or inability to drink/breastfeed and a distinct indicator variable for those cases with multiple danger signs (also coded 0 / 1). Child’s sex was also included in the adjusted regression model. Patients who died or that were discharged within 48 hours were excluded from analyses of associations with monitoring frequency because we did not have data on their length of stay in hours. The number of vital signs observations would be related to the actual length of stay for those not completing a full 48-hour stay in hospital, potentially confounding analyses of associations of patient characteristics with counts of monitoring episodes over 48 hours.

### Handling missing data

Before fitting regression models we explored the levels of missingness both in explanatory and dependent variables. While level of missingness in some of the explanatory variables was not of concern (<1%) for the primary outcome of vital signs count 20.3% cases had data on any one of the T, P or R measurements missing / not recorded. We therefore used multiple imputation to address missingness for individual T, P and R counts using chained equations [28,29] under the assumption of missing at random (MAR) before computing the vital signs count for each case. The imputation models included both key variables (as used in the analysis model) and auxiliary variables (other relevant variables included to add extra information to the imputation model as is recommended [29]). Key variables included: gender, age group, hospital identity, severe childhood illness, danger signs, and survival status of a patient. Auxiliary variables included: number of nurses per shift, ward bed capacity, temperature readings at admission, and season. Since the number of vital signs measurements in 48 hours at the point of data entry could take discrete values ranging from 0 to 10 for each of T, P and R, an ordered logit model [29] was deemed appropriate and hence used in the imputation. The simulation error was minimised by using 50 imputations with 100 iterations. We further assessed our imputation approach using convergence plots and plots of the marginal distribution of both imputed and observed values as is advised [29] and conducted sensitivity analyses to investigate the validity of the MAR assumption using pattern mixture models [30]. These procedures and results of these analyses are presented in **Online Supplementary Document(Online Supplementary Document)**. We concluded that the imputation was satisfactory and our assumption of MAR reasonable.

### Model specification

We used mixed effects models to account for clustering with hospitals included as random effects in all regression models. As vital signs counts take discrete, nonnegative values and because the Poisson model assumption of equidispersion was violated, analyses employed negative binomial regression models that account for over-dispersion. Analyses were first conducted for each of the explanatory variables to examine associations with vital signs count. All variables were then included in multivariable mixed effects regression models with clinically relevant interactions explored using likelihood ratio tests. No interactions were found to be significant and results indicated that multi-collinearity was not a concern. Both univariate and multivariable models were fitted using 50 multiply imputed data sets. Visual inspection of residual plots did not show obvious departures from the model assumptions. We therefore derived final estimates of the mixed effects negative binomial regression models (univariate and multivariable) pooled from all multiply imputed data sets using Rubin’s rules [31]. We conducted a sensitivity analysis to explore the consistency of our estimates as follows; we fitted our final model using a data set (n = 42 500) where patients with missing data on their outcome (alive/dead) had been multiply imputed using chain equations. The estimates for associations from these sensitivity analyses were not appreciably different from those we report using the data set (n = 41 738) that excluded patients without outcome (alive/dead) data. All analyses were performed using R Version 3.2.5 (R Foundation for Statistical Computing, Vienna, Austria; http://www.cran.r-project.org).

## RESULTS

### Population characteristics

The eligible study population consisted of 54 800 patients across 13 CIN hospitals from the period September 2013 through April 2016 and is described in **Table 1**. The median age of the study population was 20 months (IQR 10-48) and 54.6% were male. Overall inpatient mortality was approximately 6.1% but proportions varied considerably across hospitals (range 2.3-10.3%). The median number of beds per pediatric ward was 35 (IQR 32-41) that were attended by a median number of 2 (range 1-2) fully qualified nurses per shift. Hospital H5 with mortality approximately 3% had the highest median vital signs count of 30 while hospital H10 with a mortality of approximately 10.3% had the lowest median count of 2 for those with hospital stays of ≥48 hours (**Table 1**).

**Table 1:** Characteristics of hospitals under study

Hospital	Ward bed capacity	Nurses per shift	Malaria prevalence	Total admissions	Median age (IQR) months	Inpatient mortality (%)	
H1*	32	1	High	5030	26(13-54)	352/5030 (7.00)
H2	63	2	Low	4487	15 (8-32)	252/4487 (5.62)
H3*	35	2	High	7813	30(13-60)	597/7813 (7.64)
H4	38	1	Low	2785	18(9-35)	63/2785 (2.26)
H5	29	2	Low	3333	18(9-34)	103/3333 (3.09)
H6	67	2	Low	3613	13(7-26)	157/3613 (4.35)
H7	29	2	High	3782	30(12-60)	250/3782 (6.61)
H8	38	1	High	5723	24(11-60)	384/5723 (6.71)
H9	35	2	Low	3407	16(8-36)	192/3407 (5.64)
H10*	41	1	Low	3826	13(7-33)	392/3826 (10.25)
H11*	42	2	Low	3816	12(7-26)	292/3816 (7.65)
H12	32	1	Low	3473	19(10-38)	81/3473 (2.33)
H13	21	2	High	3712	34(16-60)	215/3712 (5.79)
Total				54800	20(10-48)	3330/54 800 (6.08)

IQR – interquartile range

*Denotes hospital with high mortality (≥7%).

### Vital signs at admission

**Figure 2:**
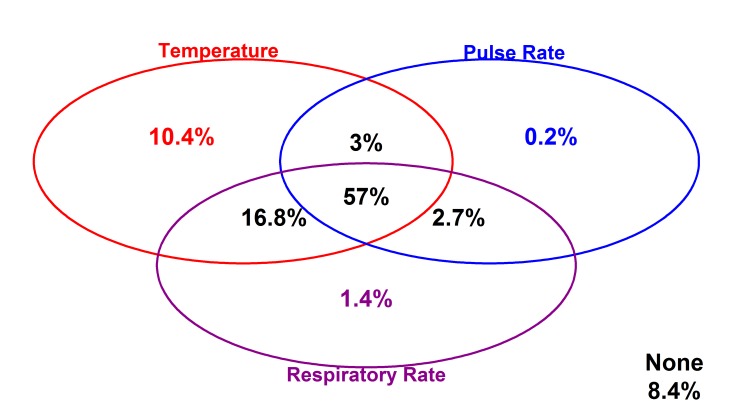
Proportion of children whose vital signs were documented at admission are represented by the oval shapes. Intersections of the ovals represent proportions of children who had either 2 or all 3 vital signs documented at admission while sections of the ovals without intersection represent proportions of children who had only 1 of the three vital signs documented. Proportion of children who had none of three vital signs documented are presented as “None”.

Clinicians recorded a full set of vital signs (TPR) for 57% of children on admission, 74% of whom were reported to have fever, while in 8.4% none of the vital signs were documented. It was more common to have a single temperature observation (10.4% of admissions) or a combination of temperature and respiratory rate observations (16.8% of admissions) where a full set of vital signs was not recorded (**Figure 2**). Although measurement of blood pressure is also regarded as a key vital sign in sick children we abandoned collecting these data as it was recorded in fewer than 2% admissions.

### Vital Signs Count variability

**Figure 3:**
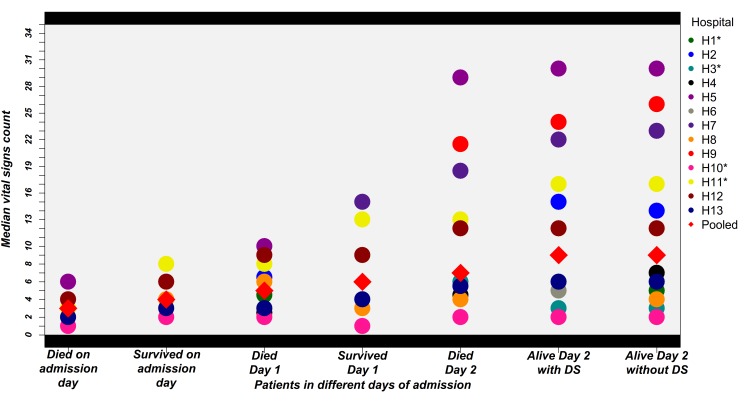
Variability of vital signs count in different populations in the first 48 hours of admission across hospitals under study. Each dot is the median vital sign count for individual hospitals in a specific population and the diamond is the median of these medians. Asterisk (*) represents hospitals of high mortality. “DS” denotes danger signs.

**Figure 4:**
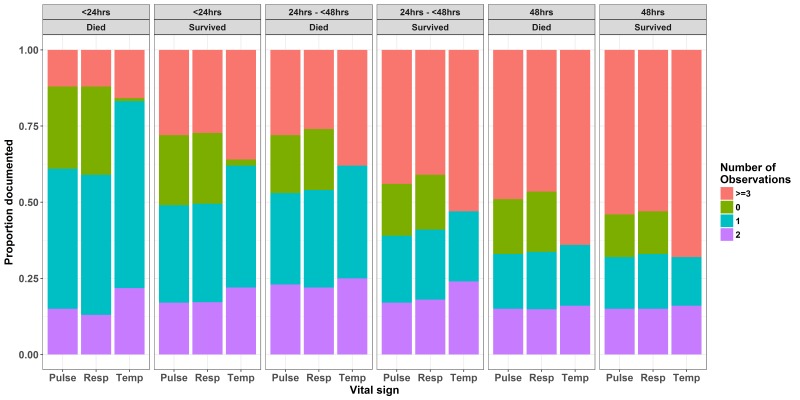
Distribution of proportions of the number of times each vital sign (T, R, P) was monitored by nurse(s) in different populations(alive/dead) across time within 48-hour period

Variability in the vital signs count across different hospitals for different populations is illustrated in **Figure 3**. As expected counts were lower in those dying on the day of admission than those who were discharged alive on the day of admission. This trend was consistent for the patients who died and who survived for 24 hours. For patients who died two days after admission the median of all 13 hospitals’ vital signs counts was still below the recommended 24-hour standard of 9. Amongst those surviving at least 48 hours the median of all 13 hospitals’ vital signs counts for patients with danger signs at admission was 9 (range of hospital medians 0-30) compared with a recommended 18 and this estimate did not differ for populations with and without danger signs. Some hospitals (H5, H7 and H9) achieved consistently high vital signs counts. Thus, surpassing the recommended 18 in 48 hours while others were consistently low (eg, H3 and H10). Exploring the counts for individual vital signs (**Figure 4**) suggests that pulse and respiratory rate measurements were more likely to be left undone across time with almost half of children having had either none, one or two pulse and respiratory rate measurements over the first 48 hours (compared with an expected count of 6). Further between 15 and 25% children who died on the admission day or the day after admission had no pulse or respiratory rate observations recorded (**Figure 4**).

### Validity of vital signs observations: digit preference

**Figure 5:**
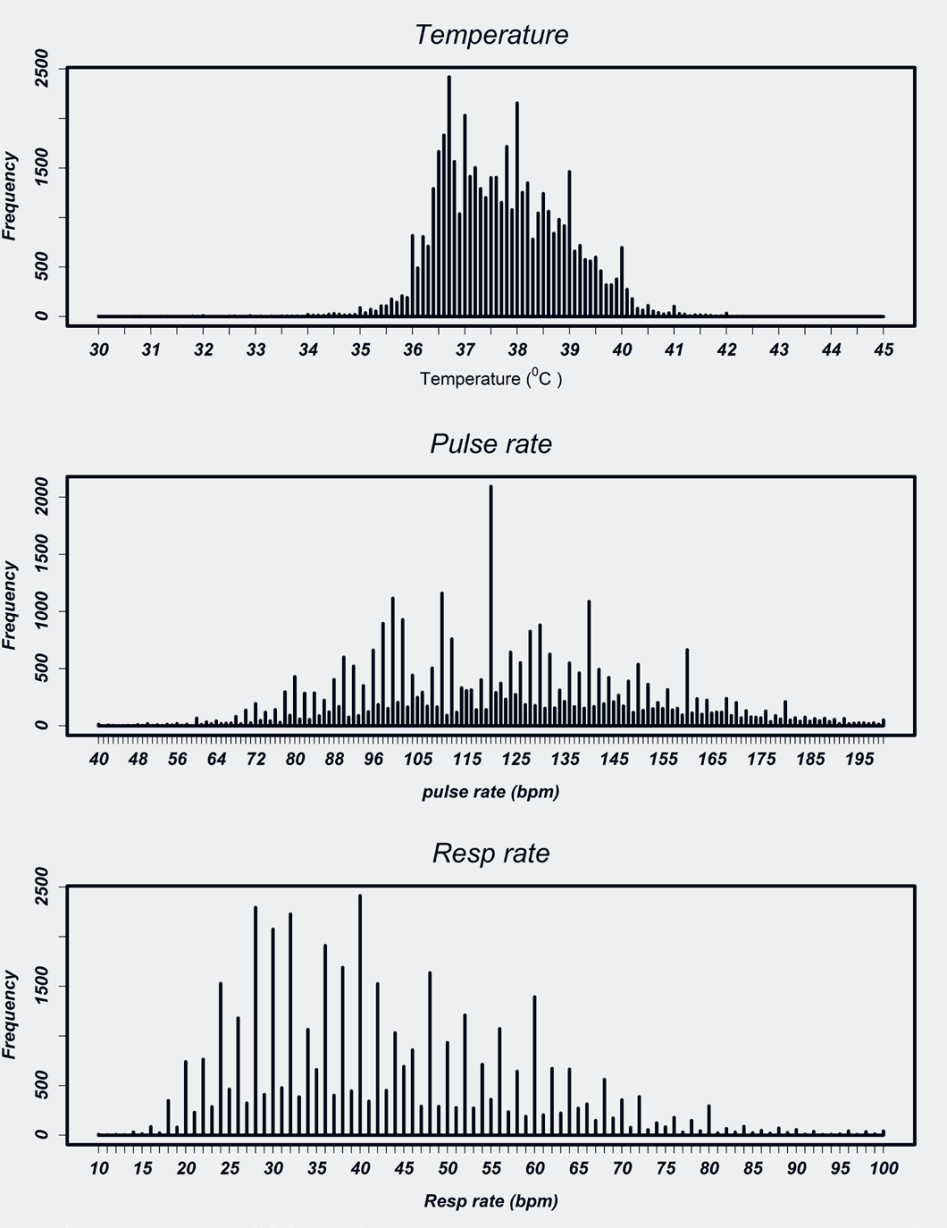
Distribution of individual vital signs readings at admission pooled across all hospitals.

Plots for vital signs values recorded at admission for each hospital showed a similar pattern. As a result, we present data pooled across hospitals (**Figure 5**). Visual inspection of the distribution of individual vital signs readings for respiratory rate and pulse rate showed marked peaks. For instance, reported readings of 40 and 120 for respiratory rate and pulse rate, respectively, were very common. There was no evidence for even/odd end-digit preference in temperature readings although there were clear peaks at values of 36.7°C, 37.0°C, 38.0°C, 39.0°C and 40.0°C.

Respiratory and pulse rate readings showed a high prevalence of even end-digits, approximately 79% and 77% respectively (see **Online Supplementary Document(Online Supplementary Document)**) that is highly unlikely to have arisen by chance (right-tailed binomial exact test in both cases *P* < 0.001).

### Multivariable analysis

In the population of patients included in multivariable analysis (**Figure 1**, n = 41 738) higher vital signs counts were weakly associated with the presence of multiple severe illnesses in a patient (adjusted risk ratio (aRR) of 1.04 (95% CI = 1.02– 1.06), *P* < 0.01), but not with any individual severe illness diagnosis. Amongst the danger signs that we considered, severe pallor was the only sign that was significantly associated with an increase of 5% in the vital signs count (aRR = 1.05 (95% CI = 1.01– 1.09), *P* = 0.02). There was no evidence to suggest a relationship between patients’ age, gender or outcome after Day 2 of admission with vital signs count (**Table 2**). The association between frequency of monitoring of each vital sign (T, P, R) considered as separate dependent variables and potential explanatory covariables was consistent with the pattern observed for the summary vital signs count (Table S1, S2 and S3 in **Online Supplementary Document(Online Supplementary Document)**).

**Table 2:** Mixed effects univariate and multivariable models’ result; Relative risk (RR) ratios, standard errors (SE) and associated 95% confidence intervals (CI) for all predictors in the analysis

			Univariate Analysis			Multivariable Analysis			
	**Fixed-effect parameter**	**RR(SE)**		**95% CI**	***P-value***		**aRR(SE)**	**95% CI**	***P-*value**
Severe illness	Low risk illness	ref		ref	ref		ref	ref	ref
	Severe malaria	0.98 (0.01)		0.95, 1.01	0.16		0.98 (0.01)	0.96, 1.01	0.16
	Meningitis	1.00 (0.02)		0.97, 1.04	0.79		1.00 (0.02)	0.97, 1.04	0.91
	Severe pneumonia	1.00 (0.01)		0.99, 1.02	0.55		1.00 (0.01)	0.99, 1.02	0.69
	Severe anemia	1.01 (0.03)		0.95, 1.07	0.71		0.99 (0.03)	0.93, 1.05	0.76
	Severe dehydration	1.00 (0.02)		0.97, 1.04	0.81		1.00 (0.02)	0.97, 1.04	0.86
	Severe malnutrition	1.03 (0.02)		0.99, 1.07	0.15		1.03 (0.02)	0.99, 1.07	0.11
	Multiple severe illness	1.04 (0.01)		1.02, 1.06	<0.01		1.04 (0.01)	1.02, 1.06	<0.01
Danger sign	No danger sign	ref		ref	ref		ref	ref	ref
	Acidotic breathing	1.08 (0.04)		1.00, 1.17	0.05		1.08 (0.04)	1.00, 1.17	0.06
	Convulsed	1.01 (0.01)		0.99, 1.02	0.58		1.01 (0.01)	0.99, 1.03	0.36
	Cyanosis	0.91 (0.09)		0.77, 1.08	0.29		0.91 (0.09)	0.77, 1.08	0.28
	Grunting	1.02 (0.01)		1.00, 1.05	0.09		1.02 (0.01)	0.99, 1.05	0.12
	Not alert	1.05 (0.04)		0.97, 1.13	0.21		1.04 (0.04)	0.97, 1.12	0.27
	Severe pallor	1.06 (0.02)		1.02, 1.11	<0.01		1.05 (0.02)	1.01, 1.09	0.02
	Unable to drink	0.99 (0.02)		0.96, 1.02	0.35		0.98 (0.02)	0.95, 1.02	0.31
	Vomit everything	1.01 (0.01)		0.99, 1.03	0.51		1.01 (0.01)	0.99, 1.03	0.43
	Multiple danger signs	1.01 (0.01)		1.00, 1.03	0.10		1.01 (0.01)	0.99, 1.03	0.35
Age group	1-11 months	ref		ref	ref		ref	ref	ref
	12-59 months	0.99 (0.01)		0.97, 1.00	0.04		0.99 (0.01)	0.97, 1.00	0.05
	≥60 months	1.00 (0.01)		0.99, 1.02	0.60		1.01 (0.01)	0.99, 1.03	0.51
Outcome≥Day 2	Died	ref		ref	ref		ref	ref	ref
	Alive	0.99 (0.02)		0.95, 1.03	0.60		1.00 (0.02)	0.97, 1.04	0.87
Gender	Female	ref		ref	ref		ref	ref	ref
	Male	1.00 (0.01)		0.98, 1.01	0.51		1.00 (0.01)	0.98, 1.01	0.48

aRR – adjusted relative risk ratio, SE – standard error, CI – confidence interval

## DISCUSSION

We used data from 13 hospitals in Kenya, over a period of more than 2 years, to describe recording of vital signs in over 54 000 children at admission by clinicians and also explored subsequent vital signs monitoring by nurses in over 41 000 children surviving at least 48 hours in the paediatric wards. Mortality in the populations studied varied from 2% to 10% across hospitals and in 9/13 was greater than 5%, a value probably higher than found in many paediatric high dependency or even intensive care units in high income settings [32]. In such high risk populations, a significant proportion of children (43%) did not have their temperature, respiratory rate and pulse rate recorded in the clinician’s medical record at admission. This suggests clinicians themselves should take a more active role in ensuring vital signs are measured and recorded at admission. There is debate over the frequency with which vital signs should be monitored particularly in critical care, telemetry units and progressive care [33] and the thresholds identifying potential concerns that should prompt action [9,18,20,34]. Such monitoring may help prevent deterioration and poor outcomes when interpreted carefully and combined with the review of clinical status that their performance encourages [15,16,35]. Across the world vital signs monitoring is therefore an accepted standard of care for sick patients, something echoed in consensus discussions with senior nurses in the hospitals studied. To evaluate adherence to the agreed standard we did not collect data on the actual values of vital signs recorded by nurses in the first 48 hours of admission. Instead we simply counted the number of times a measure was documented in the nurses’ vital signs’ charts and compared this with a locally agreed standard of 3 times in each 24 hours. Consistently high vital signs counts were seen in 3/13 hospitals but overall across hospitals counts were low compared with the local agreed standard of care (**Figure 3**). In these hospitals each qualified nurse is typically responsible for between 10 and 41 beds on a ward (median 31) and there can be 2 or more children admitted to a bed. In 5 of 13 hospitals typically only one qualified nurse is present on a shift and in the other 8 there are typically only two. It is perhaps not surprising therefore that vital signs may be left unmeasured or unrecorded and that there was little evidence of nurses prioritizing sicker patients for monitoring. A study conducted in Kenya which primarily focused on neonatal care services provided by internship training facilities also highlighted poor documentation of vital signs as one of the challenges limiting the quality of clinical care [36]. Missed monitoring may also be because it is considered time consuming and overwhelming [37] or a low priority task [38]. However, it is hard to ignore the likely effect of high patient to nurse ratios. Although, human resource shortages in general have been highlighted elsewhere [39,40] our data point to the practical impact of nursing shortages. As missed care and lower nurse to patient ratios are associated with each other and poor outcomes in high income settings [41,42] tackling the stark deficiencies in nursing staffing seen in these Kenyan settings would appear to be a high priority. Greater attention to benchmarks for nursing and minimum staff-patient ratios for hospitals might improve the situation for nurses in lower income settings.

Temperatures are still often measured with mercury thermometers in Kenyan hospitals although digital devices are becoming increasingly common. This perhaps explains why although measures of 36.7°C and 37.0°C (“normal”) and 38°C and 39°C (“high” and “very high”) were frequent there was a reasonable distribution of values. In contrast the accuracy of pulse and respiratory rate measures may be questioned. In Kenyan hospitals these counts are almost always conducted manually. Common values of pulse and respiratory rate included multiples of 10 beats/min (especially 120) and multiples of 4 and 10 breaths per minute respectively (a pattern repeated across hospitals). In both cases there was pronounced end digit preference for even numbers. While the clinical significance of the accuracy of pulse measurements might be debated the importance of accurate respiratory rates has been emphasized in WHO guidelines for assessment of the sick child for more than 30 years. In both WHO and Kenyan guidance it is advised that respiratory rates are counted for 1 minute (therefore making odd and even number counts equally likely) as this is a key part of diagnosing childhood pneumonia (an analysis of respiratory rate in those with pneumonia showed the same pattern as that for all patients shown in this paper; see **Online Supplementary Document(Online Supplementary Document)**). Our data therefore raise concerns that in practice inaccurate respiratory rate measures are likely to result in misclassification of pneumonia, poor targeting of treatments and inability to detect deterioration or improvement.

Our data illustrate a significant global paradox. Millions of individuals in high income countries now monitor their own vital status, tracking changes and sharing their data with technology companies. Generous funds have been made available to spur development of cheap, robust patient monitoring devices that should alleviate the burden of vital signs (and other) monitoring tasks undertaken by health workers in low and middle income countries. These funds have resulted in new university departments for innovation, not for profit enterprises and private businesses. Yet implementation of even basic technologies lags far, far behind and whether new technologies benefit patients or health workers in routine settings is rarely examined in low income countries [43]. A focus just on technologies also risks us ignoring the vital complementary role of health workers. This major failure in prioritization particular with regard to nurses has recently been highlighted [44], and it remains to be seen if greater investment (eg, in recruitment of new staff) will occur in LIC. In one of the few studies to explore methods to improve vital signs measurement in a low-income setting Olson et al. [45] examined implementation of the Inpatient Triage, Assessment and Treatment (ITAT) system in Malawi. Interestingly to facilitate this they used task shifting to a new cadre of trained “vital sign assistants” to help overcome workforce deficits.

### Limitations and strengths of the study

Our study has a number of limitations. Data were collected after patient discharge from medical records. We therefore capture only what is documented. However, we have developed rigorous procedures for such record review over a period of years [46] and employ timely data quality checking procedures [24]. Nurses may not document observations actually collected on the vital signs chart, and we encountered moderate levels of missingness when trying to construct a vital signs index. However, senior nurses agreed that the vital signs chart is the primary clinical record for observations as this is the tool used to share information across nursing and medical teams. To account for missingness we used multiple imputation to ensure maximum use of available data examining the robustness of this approach. The hospitals included in the CIN are also not a statistically representative sample of such facilities in Kenya, they have varying case mix [1] and over time have received regular feedback on their performance (although there has been little attention paid to nursing observations to date) [24]. Our results may therefore not be generalizable to other settings. They are also centres providing experiential training for junior or student clinicians and nurses. The latter often augment staffing on wards in support of qualified nurses. This may mean that the results we report are better than might be found in many non-training centre hospitals in Kenya or possibly worse if students record vital signs inaccurately.

## CONCLUSIONS

Previous work on quality of care in low-income countries has largely examined care provided by medical personnel or nurses delivering primary or obstetric care. We report what we believe is the largest study to date of one element of hospital based nursing practice from Africa. Although we focused on one quite specific indicator we believe the results show that efforts to improve quality and outcomes of admission may be highly dependent on improving nursing care. This will require specific attention potentially spanning better training and supervision, better prioritization of patients at risk, and most importantly addressing the inadequacies in nursing numbers. Such efforts will likely be required to enable the benefits of technologies that support patient care to be realized.

## References

[1] Ayieko P, Ogero M, Makone B, Julius T, Mbevi G, Nyachiro W (2016). Characteristics of admissions and variations in the use of basic investigations, treatments and outcomes in Kenyan hospitals within a new Clinical Information Network.. Arch Dis Child.

[2] Ogunlesi T (2015). Mortality within the first 24 hours of admission among neonates aged less than 24 hours in a special care baby unit (SCBU) in Nigeria: the role of significant hypothermia and hypoglycemia.. Iranian Journal of Neonatology..

[3] English M, Ngama M, Musumba C, Wamola B, Bwika J, Mohammed S (2003). Causes and outcome of young infant admissions to a Kenyan district hospital.. Arch Dis Child.

[4] Ayieko P, Okiro EA, Edwards T, Nyamai R, English MD (2012). . Variations in mortality in children admitted with pneumonia to Kenyan hospitals.. PLoS One.

[5] Irimu GW, Greene A, Gathara D, Kihara H, Maina C, Mbori-Ngacha D (2014). Factors influencing performance of health workers in the management of seriously sick children at a Kenyan tertiary hospital–participatory action research.. BMC Health Serv Res.

[6] Jones A; Royal Prince Alfred Hospital Patient Observation. (Vital Signs) Policy - Adult, in Policy Directive. 2010, Sydney South West Area Health Service (SSWAHS). p. 1-13.

[7] Asiimwe SB, Okello S, Moore CC (2014). Frequency of vital signs monitoring and its association with mortality among adults with severe sepsis admitted to a general medical ward in Uganda.. PLoS One.

[8] Hands C, Reid E, Meredith P, Smith GB, Prytherch DR, Schmidt PE (2013). Patterns in the recording of vital signs and early warning scores: compliance with a clinical escalation protocol.. BMJ Qual Saf.

[9] Miltner RS, Johnson KD, Deierhoi R (2014). Exploring the frequency of blood pressure documentation in emergency departments.. J Nurs Scholarsh.

[10] McQuillan P, Pilkington S, Allan A, Taylor B, Short A, Morgan G (1998). Confidential inquiry into quality of care before admission to intensive care.. BMJ.

[11] Lighthall GK, Markar S, Hsiung R (2009). Abnormal vital signs are associated with an increased risk for critical events in US veteran inpatients.. Resuscitation.

[12] Van Kuiken D, Huth MM (2016). What is ‘normal?’ Evaluating vital signs.. Nephrol Nurs J.

[13] Chester JG, Rudolph JL (2011). Vital signs in older patients: age-related changes.. J Am Med Dir Assoc.

[14] Watkins T, Whisman L, Booker P (2016). Nursing assessment of continuous vital sign surveillance to improve patient safety on the medical/surgical unit.. J Clin Nurs.

[15] Smith GB (2010). In-hospital cardiac arrest: is it time for an in-hospital ‘chain of prevention’?. Resuscitation.

[16] Cretikos MA, Bellomo R, Hillman K, Chen J, Finfer S, Flabouris A (2008). Respiratory rate: the neglected vital sign.. Med J Aust.

[17] Hillman KM, Bristow PJ, Chey T, Daffurn K, Jacques T, Norman SL (2001). Antecedents to hospital deaths.. Intern Med J.

[18] Barfod C, Lauritzen MM, Danker JK, Sölétormos G, Forberg JL, Berlac PA (2012). Abnormal vital signs are strong predictors for intensive care unit admission and in-hospital mortality in adults triaged in the emergency department - a prospective cohort study.. Scand J Trauma Resusc Emerg Med.

[19] Goldhill DR, McNarry AF, Mandersloot G, McGinley A (2005). A physiologically-based early warning score for ward patients: the association between score and outcome.. Anaesthesia.

[20] Egdell P, Finlay L, Pedley DK (2008). The PAWS score: validation of an early warning scoring system for the initial assessment of children in the emergency department.. Emerg Med J.

[21] Seiger N, van Veen M, Almeida H, Steyerberg EW, van Meurs AHJ, Carneiro R (2014). Improving the Manchester Triage System for pediatric emergency care: an international multicenter study.. PLoS One.

[22] Ministry of Health (MOH). Kenya. Paediatric Admitting Record Form. 2015. Available: http://www.idoc-africa.org/images/documents/Paeds%202_a_-%20PAR%20Paediatric%20Admitting%20Record%20Form.pdf. Accessed: 22 November 2016.

[23] Harris PA, Taylor R, Thielke R, Payne J, Gonzalez N, Conde JG (2009). Research electronic data capture (REDCap)–a metadata-driven methodology and workflow process for providing translational research informatics support.. J Biomed Inform.

[24] Tuti T, Bitok M, Paton C, Makone B, Malla L, Muinga N (2016). Innovating to enhance clinical data management using non-commercial and open source solutions across a multi-center network supporting inpatient pediatric care and research in Kenya.. J Am Med Inform Assoc.

[25] Ministry of Health (MOH). Kenya. Basic Paediatric protocols for ages up to 5 years. 2016. Available: http://www.idoc-africa.org/index.php/en/98-about-us/148-basic-paediatric-protocols-2016. Accessed: 22 November 2016.

[26] World Health Organization. Dept. of Child and Adolescent Health, Handbook IMCI: integrated management of childhood illness. Geneva: World Health Organization; 2005.

[27] Van den Bruel A, Thompson M, Buntinx F, Mant D (2012). Clinicians’ gut feeling about serious infections in children: observational study.. BMJ.

[28] White IR, Royston P, Wood AM (2011). Multiple imputation using chained equations: Issues and guidance for practice.. Stat Med.

[29] Buuren SV, Groothuis-Oudshoorn K (2011). mice: Multivariate imputation by chained equations in R.. J Stat Softw.

[30] Bell ML, Fairclough DL (2014). Practical and statistical issues in missing data for longitudinal patient-reported outcomes.. Stat Methods Med Res.

[31] Rubin DB. Multiple imputation for nonresponse in surveys. Vol. 81. John Wiley & Sons: Oxford; 2004.

[32] Pollack MM, Holubkov R, Funai T, Berger JT, Clark AE, Meert K (2015). Simultaneous prediction of new morbidity, mortality, and survival without new morbidity from pediatric intensive care: a new paradigm for outcomes assessment.. Crit Care Med.

[33] Schulman CS, Staul L (2010). Standards for frequency of measurement and documentation of vital signs and physical assessments.. Crit Care Nurse.

[34] Ayodele OE, Sanya EO, Okunola OO, Akintunde AA (2012). End digit preference in blood pressure measurement in a hypertension specialty clinic in southwest Nigeria.. Cardiovasc J Afr.

[35] Fieselmann JF, Hendryx MS, Helms CM, Wakefield DS (1993). Respiratory rate predicts cardiopulmonary arrest for internal medicine inpatients.. J Gen Intern Med.

[36] Aluvaala J, Nyamai R, Were F, Wasunna A, Kosgei R, Karumbi J (2015). Assessment of neonatal care in clinical training facilities in Kenya.. Arch Dis Child.

[37] Mok W, Wang W, Cooper S, Ang EN, Liaw SY (2015). Attitudes towards vital signs monitoring in the detection of clinical deterioration: scale development and survey of ward nurses.. Int J Qual Health Care.

[38] Luettel D, Beaumont K, Healey F. Recognising and responding appropriately to early signs of deterioration in hospitalised patients. 2007, National Patient Safety Agency (NPSA). Available: http://www.nrls.npsa.nhs.uk/resources/?entryid45=59834. Accessed: 22 November 2016.

[39] Chen L, Evans T, Anand S, Boufford JI, Brown H, Chowdhury M (2004). Human resources for health: overcoming the crisis.. Lancet.

[40] Wakaba M, Mbindyo P, Ochieng J, Kiriinya R, Todd J, Waudo A (2014). The public sector nursing workforce in Kenya: a county-level analysis.. Hum Resour Health.

[41] Jones TL, Hamilton P, Murry N (2015). Unfinished nursing care, missed care, and implicitly rationed care: State of the science review.. Int J Nurs Stud.

[42] Watson SI, Arulampalam W, Petrou S, Marlow N, Morgan AS, Draper ES (2016). The effects of a one-to-one nurse-to-patient ratio on the mortality rate in neonatal intensive care: a retrospective, longitudinal, population-based study.. Arch Dis Child Fetal Neonatal Ed.

[43] Enoch AJ, English M, Shepperd S (2016). Does pulse oximeter use impact health outcomes? A systematic review.. Arch Dis Child.

[44] All-Party Parliamentary Group on Global Health. Triple Impact – how developing nursing will improve health, promote gender equality and support economic growth. Available: http://www.appg-globalhealth.org.uk/. Accessed: 22 November 2016.

[45] Olson D, Davis NL, Milazi R, Lufesi N, Miller WC, Preidis GA (2013). Development of a severity of illness scoring system (inpatient triage, assessment and treatment) for resource-constrained hospitals in developing countries.. Trop Med Int Health.

[46] Mwaniki P, Ayieko P, Todd J, English M (2014). Assessment of paediatric inpatient care during a multifaceted quality improvement intervention in Kenyan District Hospitals–use of prospectively collected case record data.. BMC Health Serv Res.

