# Supplementary material for: An observational study of monitoring of vital signs in children admitted to Kenyan hospitals: an insight into the quality of nursing care?
Source: J Glob Health. 2018 Feb 17;8(1):010409. doi: 10.7189/jogh.08.010409 (PMC5826085; doi:10.7189/jogh.08.010409)
Supplement: Online Supplementary Document [file jogh-08-010409-s001.pdf]

Online Supplementary Document

Ogero et al. An observational study of monitoring of vital signs in children admitted to Kenyan hospitals: an insight into the quality of nursing care?

J Glob Health 2018;8:010409

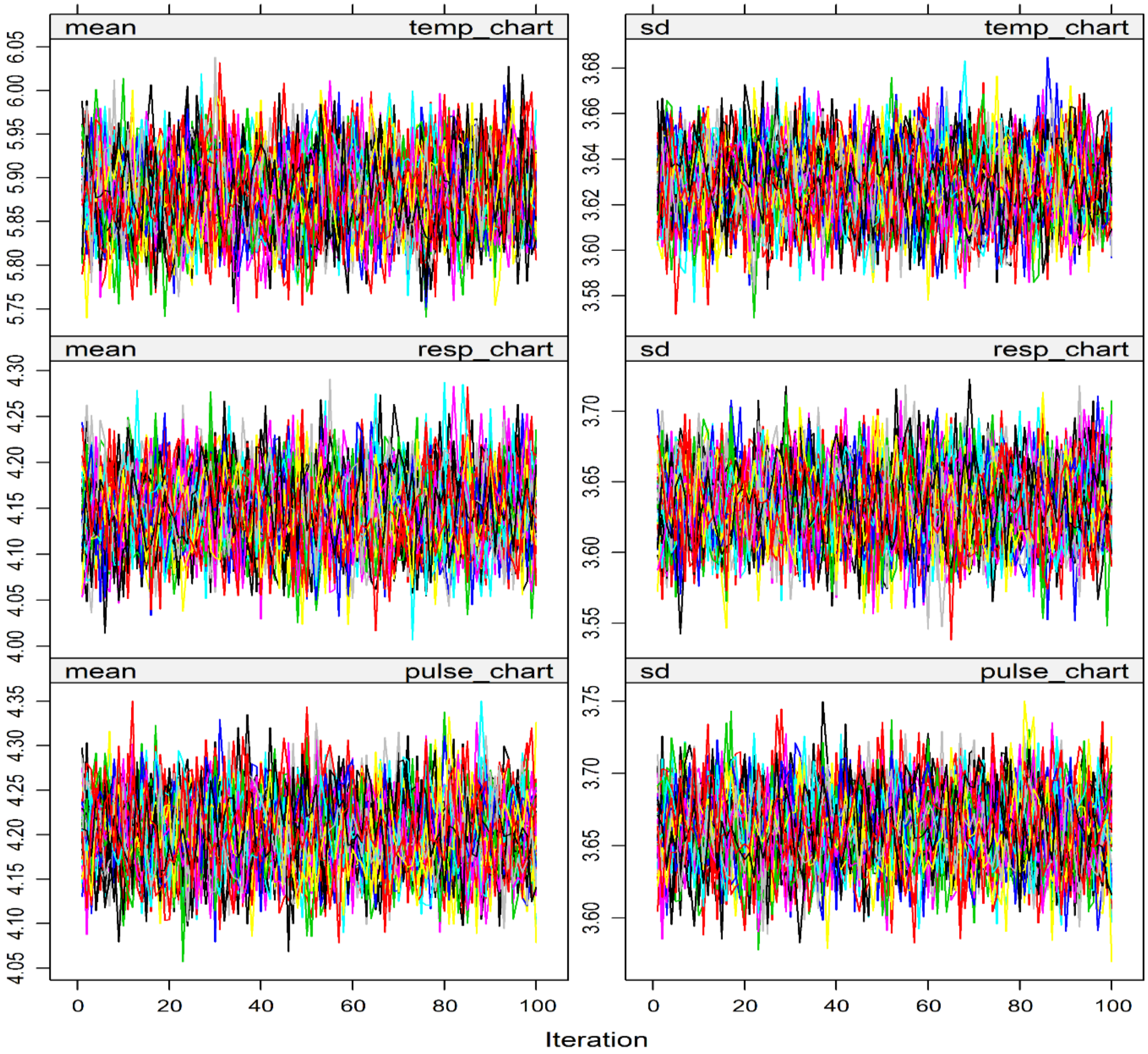

Figure S1. Diagnostic convergence plot of Multiple Imputation by Chained Equations (MICE) algorithm. Lines in the plot represents each of the 50 imputed datasets. Left and right sided panels represents means and variances respectively per iteration of the imputed values of

Temperature (represented as temp_chart), Respiratory Rate (represented as resp_chart) and Pulse Rate (represented as pulse_chart). Plots suggest the imputing algorithm converged since lines intermingled freely with each other right from the start without showing any definite trend.

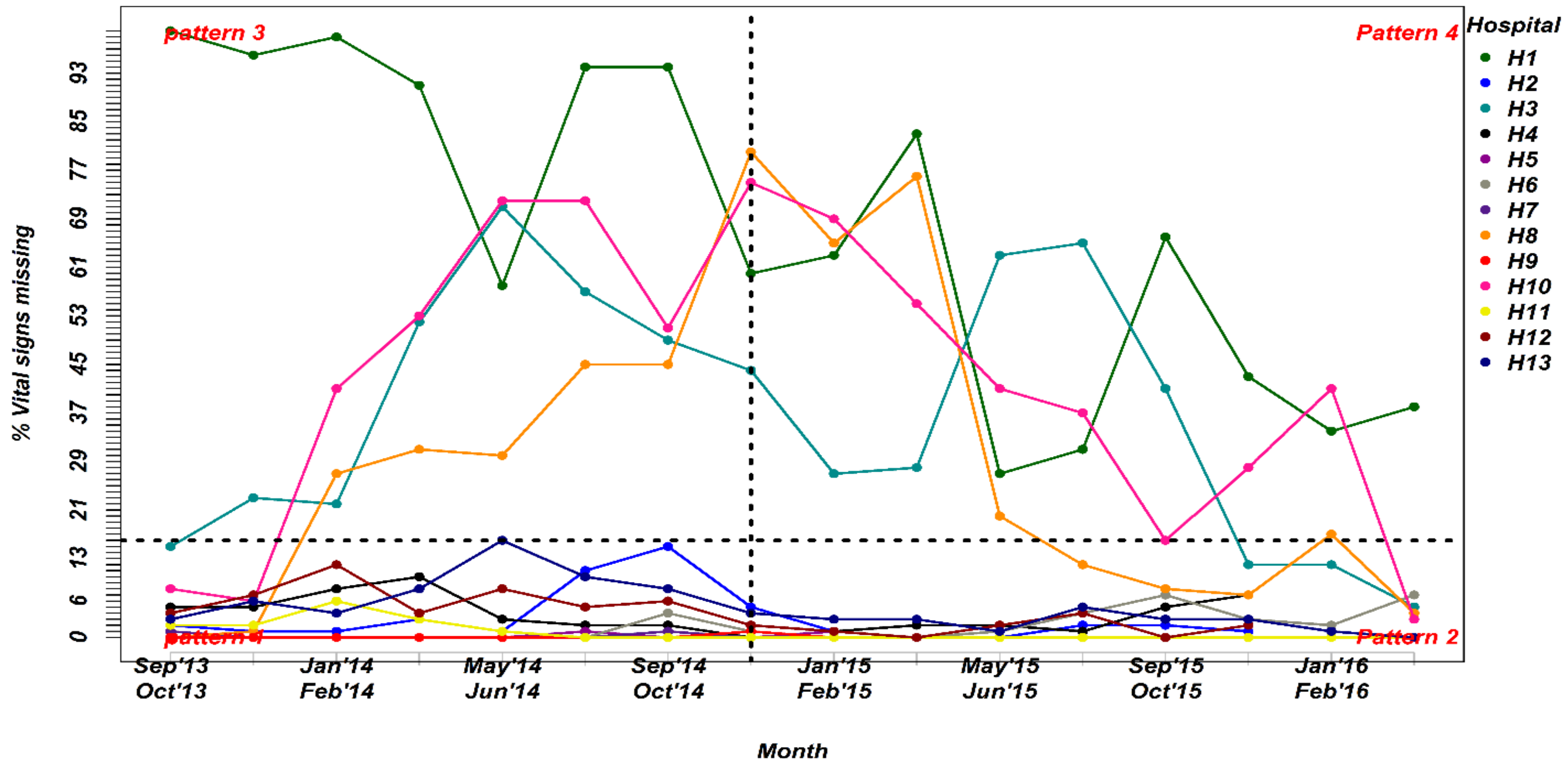

Figure S2. A plot showing the degrees of missingness of vital signs for each hospital during the study period (September 2013 – April 2016).

To demarcate different patterns of missingness, a horizontal dotted line is drawn at 16% of missingness and vertical dotted line is drawn after one year of study, December 2014. Pattern 1 (n=10,406) consisted of hospitals that had missingness below 16% within the

first year of study, pattern 2 (n=13,565) consisted of hospitals that had missingness below 16% after the first year study, pattern 3 (n=6,568) consisted of hospitals that had missingness above 16% within the first year of study, and pattern 4 (n=5,817) consisted of hospitals that had missingness above 16% after the first year of study.

The objective of this approach was to develop parameter estimates from identical models applied to datasets with different missing data patterns [S1]. Four patterns were generated based on the different levels of vital signs missingness across different hospitals and periods of time. These patterns were as illustrated in the plot presented S2 Appendix. For each dataset (pattern) we used the same imputation procedure and regression model strategies as presented in the main text. This generated four sets of model estimates. A single combined estimate of association for each covariate was then produced from across these four patterns using weights according to the proportions of observations in each data patterns [S1]. Comparison of these estimates and those found under MAR assumption were not discernibly different in terms of magnitude of the coefficients.

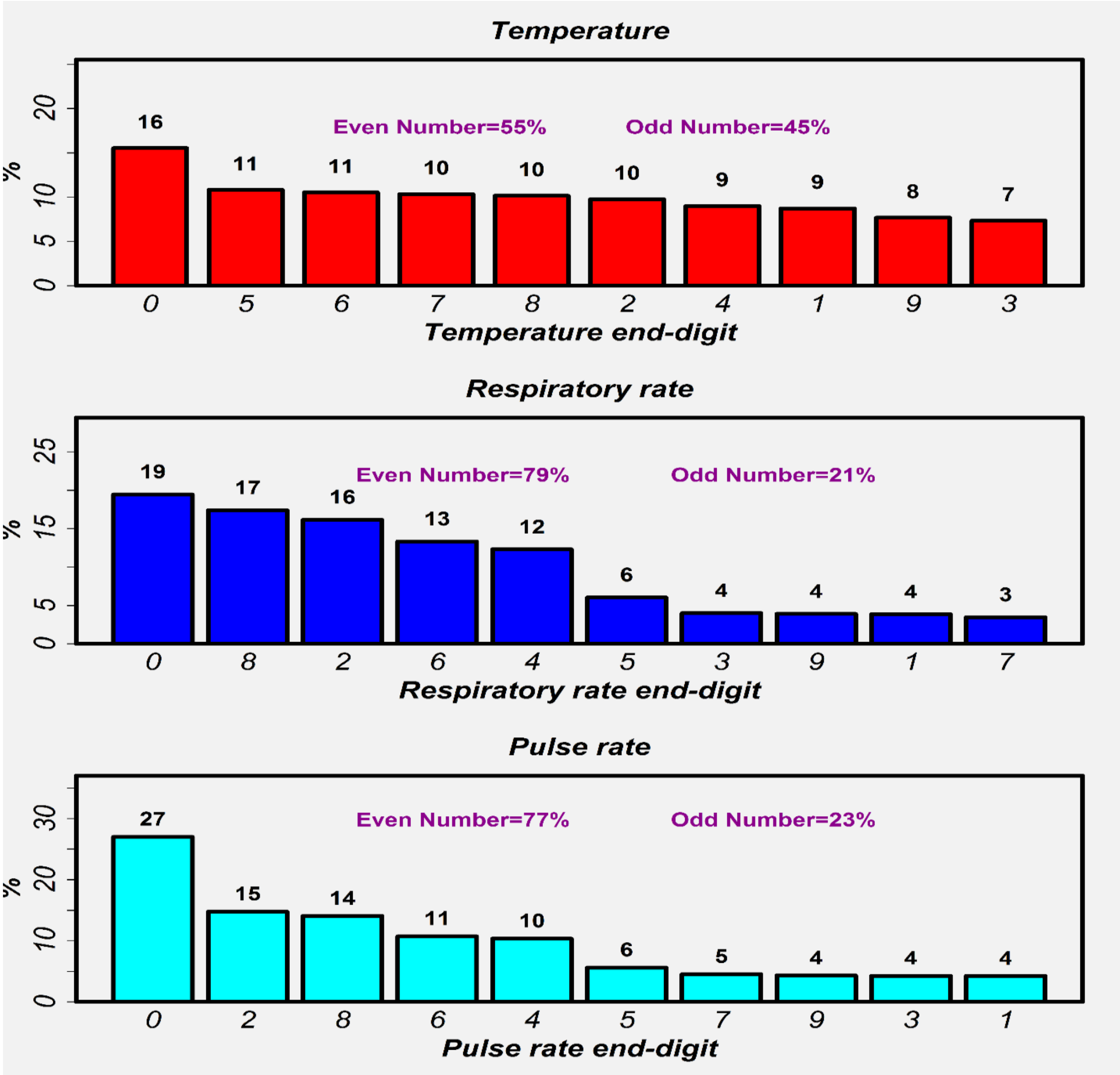

Figure S3. End-Digit preference of the vital signs readings at admission.

Each of the end numbers 0-9 is represented in the x-axis with a proportion (top of the bar) with which it appeared as an end-digit in the documented readings. Even Number and Odd Number represents a total proportion of end-digits that are even numbers and odd numbers respectively.

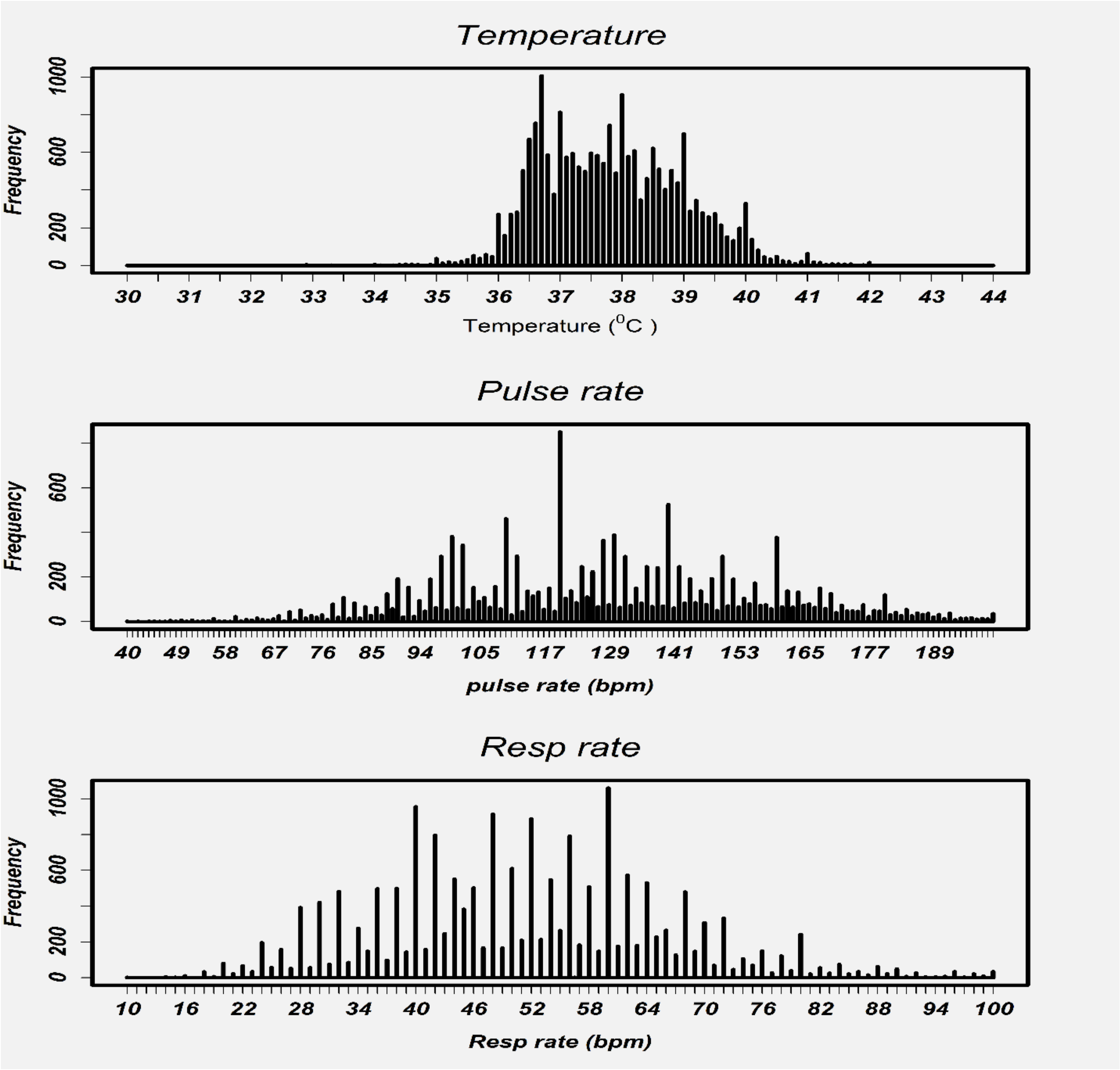

Figure S4. Distribution of individual vital signs readings at admission pooled across all hospitals for pneumonia population.

Table S1: Mixed effects univariate and multivariable models' result for temperature observations: Relative risk (RR) ratios, Standard errors (SE) and associated 95 % confidence intervals (CI) for all predictors in the analysis.

		Temperature Univariate Analysis			Temperature Multivariable Analysis		
	Fixed-Effect Parameter	RR(SE)	CI	p-value	aRR(SE)	CI	p-value
Severe Illness	Low risk illness	ref	ref	ref	ref	ref	ref
	Severe Malaria	0.99(0.01)	(0.96,1.02)	0.51	0.99(0.01)	(0.96 , 1.02)	0.48
	Meningitis	1.03(0.02)	(1.00,1.06)	0.03	1.03(0.02)	(1.00 , 1.06)	0.06
	Severe Pneumonia	1.01(0.01)	(1.00,1.03)	0.03	1.01(0.01)	(1.00 , 1.03)	0.09
	Severe Anemia	1.01(0.03)	(0.96,1.08)	0.62	1.00(0.03)	(0.94 , 1.07)	0.94
	Severe Dehydration	1.00(0.02)	(0.97,1.04)	0.81	1.00(0.02)	(0.97 , 1.04)	0.85
	Severe Malnutrition	1.04(0.02)	(1.01,1.08)	0.02	1.04(0.02)	(1.01, 0.08)*	0.02
	Multiple Severe Illness	1.04(0.01)	(1.02,1.06)	<0.0001	1.03(0.01)	(1.01, 1.05)*	<0.0001
Danger sign	No danger sign	ref	ref	ref	ref	ref	ref
	Acidotic breathing	1.05(0.04)	(0.97,1.14)	0.23	1.05(0.04)	(0.97, 1.14)	0.26
	Convulsed	1.01(0.01)	(0.99,1.02)	0.49	1.01(0.01)	(0.99, 1.03)	0.27
	Cyanosis	0.93(0.08)	(0.79,1.08)	0.34	0.92(0.08)	(0.79, 1.08)	0.33
	Grunting	1.02(0.01)	(0.99,1.04)	0.14	1.01(0.01)	(0.99, 1.04)	0.27
	Not Alert	1.02(0.04)	(0.94,1.11)	0.57	1.02(0.04)	(0.94, 1.11)	0.62
	Severe Pallor	1.04(0.02)	(1.00,1.08)	0.06	1.03(0.02)	(0.99, 1.08)	0.18
	Unable to drink	1.00(0.01)	(0.97,1.03)	0.89	1.00(0.01)	(0.97, 1.03)	0.86
	Vomit everything	1.00(0.01)	(0.98,1.02)	0.65	1.00(0.01)	(0.98, 1.02)	0.80
	Multiple Danger Signs	1.01(0.01)	(1.00,1.03)	0.07	1.01(0.01)	(1.00, 1.03)	0.18
Age group	1-11 months	ref	ref	ref	ref	ref	ref
	12-59 months	0.99(0.01)	(0.97,1.00)	0.04	0.99(0.01)	(0.98 , 1.00)	0.06
	>=60 months	0.99(0.01)	(0.98,1.01)	0.57	1.00(0.01)	(0.98 , 1.02)	0.74
Outcome ≥ Day 2	Died	ref	ref	ref	ref	ref	ref
	Alive	1.00(0.02)	(0.96,1.04)	0.98	1.01(0.02)	(0.98 , 1.05)	0.50
Gender	Female	ref	ref	ref	ref	ref	ref
	Male	0.99(0.01)	(0.98,1.00)	0.20	0.99(0.01)	(0.98 , 1.00)	0.19

Table S2: Mixed effects univariate and multivariable models' result for respiratory rate observations: Relative risk (RR) ratios, Standard errors (SE) and associated 95 % confidence intervals (CI) for all predictors in the analysis.

		Respiratory rate Univariate Analysis			Respiratory rate Multivariable Analysis		
	Fixed-Effect Parameter	RR(SE)	CI	p-value	aRR(SE)	CI	p-value
Severe Illness	Low risk illness	ref	ref	ref	ref	ref	ref
	Severe Malaria	0.97(0.02)	(0.93,1.01)	0.15	0.97(0.02)	(0.93, 1.01)	0.16
	Meningitis	0.97(0.02)	(0.92,1.02)	0.23	0.97(0.03)	(0.92, 1.02)	0.21
	Severe Pneumonia	1.00(0.01)	(0.97,1.02)	0.75	0.99(0.01)	(0.97, 1.02)	0.66
	Severe Anemia	1.02(0.04)	(0.94,1.11)	0.62	1.00(0.05)	(0.92, 1.10)	0.92
	Severe Dehydration	0.99(0.03)	(0.94,1.04)	0.65	0.99(0.03)	(0.94, 1.04)	0.70
	Severe Malnutrition	1.02(0.03)	(0.96,1.08)	0.57	1.02(0.03)	(0.96, 1.08)	0.47
	Multiple Severe Illness	1.03(0.01)	(1.00,1.06)	0.02	1.03(0.02)	(1.00, 1.06)*	0.04
Danger sign	No danger sign	ref	ref	ref	ref	ref	ref
	Acidotic breathing	1.09(0.07)	(0.96,1.25)	0.18	1.09(0.07)	(0.96, 1.24)	0.19
	Convulsed	1.00(0.01)	(0.98,1.03)	0.77	1.01(0.01)	(0.98, 1.03)	0.57
	Cyanosis	0.90(0.12)	(0.71,1.14)	0.37	0.90(0.12)	(0.70, 1.14)	0.37
	Grunting	1.03(0.02)	(0.99,1.07)	0.11	1.03(0.02)	(0.99, 1.07)	0.11
	Not Alert	1.05(0.05)	(0.94,1.17)	0.37	1.04(0.05)	(0.94, 1.16)	0.42
	Severe Pallor	1.06(0.03)	(1.00,1.12)	0.04	1.04(0.03)	(0.98, 1.11)	0.18
	Unable to drink	0.96(0.02)	(0.92,1.01)	0.10	0.96(0.02)	(0.92, 1.01)	0.10
	Vomit everything	1.01(0.02)	(0.98,1.04)	0.52	1.01(0.02)	(0.98, 1.04)	0.48
	Multiple Danger Signs	1.00(0.01)	(0.98,1.03)	0.75	1.00(0.01)	(0.97, 1.03)	0.99
Age group	1-11 months	ref	ref	ref	ref	ref	ref
	12-59 months	0.99(0.01)	(0.97,1.01)	0.19	0.99(0.01)	(0.97 , 1.01)	0.22
	>=60 months	1.01(0.01)	(0.98,1.04)	0.45	1.01(0.01)	(0.98 , 1.04)	0.41
Outcome ≥ Day 2	Died	ref	ref	ref	ref	ref	ref
	Alive	1.01(0.03)	(0.96,1.07)	0.69	1.02(0.03)	(0.97 , 1.08)	0.44
Gender	Female	ref	ref	ref	ref	ref	ref
	Male	1.00(0.01)	(0.98,1.02)	0.94	1.00(0.01)	(0.98 , 1.02)	0.90

Table S3. Mixed effects univariate and multivariable models' result for pulse rate observations: Relative risk (RR) ratios, Standard errors (SE) and associated 95 % confidence intervals (CI) for all predictors in the analysis.

		Pulse rate Univariate Analysis			Pulse rate Multivariable Analysis		
	Fixed-Effect Parameter	RR(SE)	CI	p-value	aRR(SE)	CI	p-value
Severe Illness	Low risk illness	ref	ref	ref	ref	ref	ref
	Severe Malaria	0.97(0.02)	(0.93,1.01)	0.17	0.97(0.02)	(0.93 , 1.01)	0.18
	Meningitis	0.99(0.02)	(0.95,1.04)	0.71	0.99(0.02)	(0.95 , 1.03)	0.63
	Severe Pneumonia	1.00(0.01)	(0.98,1.02)	0.90	1.00(0.01)	(0.98 , 1.02)	0.94
	Severe Anemia	1.01(0.04)	(0.93,1.09)	0.90	0.98(0.04)	(0.91 , 1.07)	0.69
	Severe Dehydration	1.01(0.03)	(0.96,1.06)	0.63	1.01(0.03)	(0.96 , 1.07)	0.60
	Severe Malnutrition	1.03(0.03)	(0.97,1.08)	0.32	1.03(0.03)	(0.98 , 1.09)	0.26
	Multiple Severe Illness	1.04(0.01)	(1.01,1.06)	0.01	1.03(0.01)	(1.00, 1.06)*	0.02
Danger sign	No danger sign	ref	ref	ref	ref	ref	ref
	Acidotic breathing	1.10(0.06)	(0.98,1.23)	0.11	1.09(0.06)	(0.98 , 1.22)	0.13
	Convulsed	1.01(0.01)	(0.98,1.03)	0.67	1.01(0.01)	(0.98 , 1.03)	0.48
	Cyanosis	0.91(0.11)	(0.74,1.13)	0.41	0.91(0.11)	(0.73 , 1.13)	0.41
	Grunting	1.03(0.02)	(1.00,1.07)	0.07	1.03(0.02)	(1.00, 1.07)	0.07
	Not Alert	1.04(0.05)	(0.94,1.15)	0.42	1.03(0.05)	(0.94 , 1.14)	0.50
	Severe Pallor	1.07(0.03)	(1.01,1.13)	0.02	1.05(0.03)	(1.00, 1.11)	0.07
	Unable to drink	0.98(0.02)	(0.94,1.02)	0.38	0.98(0.02)	(0.94, 1.02)	0.36
	Vomit everything	1.01(0.01)	(0.98,1.04)	0.54	1.01(0.01)	(0.98, 1.04)	0.53
	Multiple Danger Signs	1.01(0.01)	(0.99,1.03)	0.42	1.00(0.01)	(0.98, 1.03)	0.70
Age group	1-11 months	ref	ref	ref	ref	ref	ref
	12-59 months	0.99(0.01)	(0.97,1.01)	0.22	0.99(0.01)	(0.97, 1.01)	0.29
	>=60 months	1.01(0.01)	(0.98,1.03)	0.47	1.01(0.01)	(0.99, 1.04)	0.37
Outcome ≥ Day 2	Died	ref	ref	ref	ref	ref	ref
	Alive	1.00(0.03)	(0.94,1.06)	0.95	1.01(0.03)	(0.95, 1.07)	0.72
Gender	Female	ref	ref	ref	ref	ref	ref
	Male	1.00(0.01)	(0.98,1.01)	0.78	1.00(0.01)	(0.98, 1.01)	0.75

Reference

1. Bell, M.L. and D.L. Fairclough, *Practical and statistical issues in missing data for longitudinal patient-reported outcomes.* Stat Methods Med Res, 2014. **23**(5): p. 440-59.
